# Community health resource project: highlighting One Health resources across rural Georgia to build healthier communities

**DOI:** 10.3389/fpubh.2025.1619886

**Published:** 2025-11-06

**Authors:** Tanya E. Jules, Megan O. Mercer, Jessica S. Schwind, Patricia LaRose-Walthour, Jennifer L. Drey, Jill Johns, Michelle N. Tremblay

**Affiliations:** Institute for Health Logistics and Analytics, Georgia Southern University, Statesboro, GA, United States

**Keywords:** Participatory Asset Mapping, rural, Georgia, One Health, strengths-based, community engagement strategies

## Abstract

Public health professionals frequently engage with residents of rural Georgia to conduct needs-based initiatives, which aim to identify deficiencies and shortcomings in community health. However, this process can exacerbate existing stereotypes and lead community members to feel a sense of despair in their own communities. The Community Health Resource Project (CHRP) offers a counterbalance through a strengths-based approach by highlighting animal, plant, human, and environmental resources, or “One Health” assets, that currently exist in the community. CHRP begins by analyzing publicly available county-level data to gain an initial understanding of the health landscape before proceeding to the field. Next, the team engages in Participatory Asset Mapping (PAM) to gather community-driven qualitative insights on existing One Health assets in participating rural or underserved counties. Data gathered from community engagement strategies inform the development of comprehensive county-specific asset maps and reports. This paper describes the methods of applying a strengths-based approach to highlight community One Health-related assets. These strategies can be a valuable tool for developing targeted workforce development efforts in resource-limited counties for the benefit of all species.

## 1 Introduction

### 1.1 Background

Rural communities face unique challenges that impact residents’ abilities to access resources that promote optimal health and well-being (1). Individuals in rural areas, on average, have higher poverty rates, lower traditional education rates, and a higher prevalence of chronic diseases compared to their metropolitan counterparts (2–4). Even among rural populations, notable health disparities persist between different racial and ethnic groups (5, 6). These disparities often stem from complex systemic issues specific to rural communities that have persisted over time, including geographic isolation, limited healthcare infrastructure and access, and socioeconomic constraints (6, 7). Long travel distances to receive health services, coupled with inadequate public transportation, often result in rural community members’ lack of preventive care, delayed diagnoses, and missed treatments (8). Additionally, a limited healthcare workforce, not only in terms of primary care but also specialty care, further exacerbates the problem of achieving optimal health in rural communities (6, 9, 10).

Broader socioeconomic constraints, such as lower median incomes, higher levels of un- or under-employment, and reduced educational attainment, are all a part of the social determinants of health that impact an individual’s ability to seek, afford, and prioritize health for themselves, their households, and their communities (11, 12). At a structural level, rural communities are frequently impacted by public policy decisions that promote metropolitan-centric models that are not impactful in rural areas (13, 14). Additionally, a lack of funding allocation leads to an underinvestment in community health-promoting resources such as broadband access, health education, food availability, and behavioral health services (13, 14). This complex web of factors result in the health disparities frequently found in rural communities across the United States (5).

To address health disparities in rural designated areas in the state of Georgia, public health professionals have frequently engaged with residents to conduct needs-based initiatives or Community Health Needs Assessments (CHNAs) (10, 15, 16). CHNAs aim to develop a comprehensive understanding of a community’s health, identify deficiencies and shortcomings in health resources within communities and recommend or design further community health interventions to improve population health (17). A five-step approach is often, but not always, used in CHNAs, with identifying the community of focus as the first step (16). A review of CHNAs found that the implementation of CHNAs vary pertaining to the implementation process, participants included throughout the process, and the intended outcomes after the process is complete (17). While needs assessments can be effective in understanding gaps in health resources in order to propose possible solutions, a focus only on “the needs” may exacerbate stereotypes through evaluator bias, immediately frame problems from a place of deficit, and lead to feelings of despair in rural communities (18). Further, these initiatives may fail to adequately understand and leverage the strengths and social connections in and across community resources (18, 19). Needs-based initiatives can have the potential to worsen health disparities and obstruct the creation of sustainable, long-term solutions in rural areas (20, 21).

### 1.2 Strengths-based approach

In recent years, the focus has increasingly shifted from a needs- or deficit-based perspective towards highlighting community assets or resources through a strengths- or asset-based framework, such as Participatory Asset Mapping (PAM) (20, 21). This kind of approach is helpful in rural counties where community leadership, social cohesion, and shared values and traditions are prevalent (22). Rural counties often rely on these social networks and personal connections to access and provide support due to marginalization and a general lack of supportive services commonly found in metropolitan areas (23). Using a strengths-based approach in rural counties recognizes the resilience of rural communities and allows for the highlighting of unique ways resources are both provided and accessed (24). For example, PAM values community representation to ensure their perspectives are reflected throughout data gathering, analysis, and reporting phases (25).

Nonetheless, a strengths-based approach also relies heavily on community outreach and engagement in rural counties, especially with the dissemination of health-related information or fostering health-promoting behaviors (19, 26). If health initiatives are implemented without substantial community engagement at every stage of project development and implementation, there may be a lack of understanding of the complexities of a rural county’s needs and identities that is essential for the success of the initiatives (25). Further, identifying key stakeholders and champions for projects who can enlist support from the community members is crucial. Limiting involvement from county members may result in ineffective or inadequate interventions, unrealistic solutions, and may further rural residents’ distrust of public health interventions (27). Although members of rural communities have historically been excluded or not been prioritized from traditional research efforts, it is crucial for culturally relevant health programs to center the perspectives of rural community members (5, 28, 29). Recognizing individuals as the experts of their own communities before initiating “boots on the ground” efforts in any rural county is necessary to the participatory nature of PAM. By highlighting community-led resource identification, PAM amplifies community members’ voices and prioritizes their strengths and capabilities through their perspective (21).

Not only do individuals in rural areas receive health information differently than in metropolitan areas, but they also access non-traditional health resources uniquely from their metropolitan counterparts (25, 30). In rural communities, residents may rely on connection and non-traditional resources to positively impact health, such as mobile clinics, telehealth services, and alternative food sources (23, 30). Through a deep level of community engagement using a strengths-based approach, health professionals are able to recognize the power of various resources and their impact on health which may otherwise be elusive to individuals outside these communities (31). Interacting and engaging with community members about their sense of place and discovering key insights to make appropriate, county-specific recommendations is needed for an impactful strengths-based approach to community initiatives (27). PAM requires scaffolding on existing resources that may be diverse in nature and based outside the traditional healthcare infrastructure (21). Interpreting the highlighted resources under the One Health framework may assist the connection between non-traditional services and rural health.

### 1.3 The need for One Health

There is a dearth of literature showcasing community initiatives through a holistic lens such as One Health, which stresses the interdependence of human, plant, animal, and environmental health (32, 33). Adopted by the World Health Organization and the Centers for Disease Control and Prevention, One Health is a globally recognized collaborative approach that incorporates the interconnectedness of people, animals, plants, and our shared environment into strategies that achieve optimal health outcomes and well-being for all (34, 35). Because emerging infectious diseases, antimicrobial resistance, climate change, and food safety and security represent a significant threat to health across all populations, the One Health approach is increasingly important to our comprehensive understanding of and response to global challenges (36).

Besides limited healthcare access and utilization, rural counties often face higher rates of zoonotic disease transmission and have a closer interdependence with local ecosystems and livestock, as animals can serve as both an income source and a food source (37, 38). Because traditional CHNAs tend to operate in discipline-specific silos, it is important to not overlook the cross-cutting nature of the broader social determinants of health that transcends species’ boundaries (39). If CHNAs are conducted using a single perspective (e.g., solely for the benefit of human health) or do not incorporate systems thinking, they would be less likely to result in realistic, sustainable solutions for the benefit of rural community health and all the species that support its success (40, 41). Therefore, the complexities associated with the socio-cultural dynamics in rural counties coupled with a strong reliance on agriculture truly necessitates a One Health approach (33, 42). Incorporating the One Health approach into strengths-based initiatives in rural communities is critical to effectively recognizing and addressing the intricacies of health problems, specifically among vulnerable, medically-underserved populations (43).

### 1.4 The Community Health Resource Project

The Community Health Resource Project (CHRP) is an initiative that offers a counterbalance to CHNAs by highlighting the strengths of One Health resources in each community through PAM. CHRP centered community engagement while catalyzing sustainable collaborative partnerships for asset identification. CHRP further centered community engagement through the lens of One Health, relying on community outreach to influence the highlighting and county-specific understanding of the inextricable interdependence of human, plant, animal, and environmental health. CHRP employed this strengths-based, holistic approach to honor the importance of connection in rural community identity, as well as fully integrate the social determinants of health. This approach allowed for the team to develop community-driven workforce development recommendations influenced by strengths-based conversations with experts in each county.

### 1.5 Project objectives

The CHRP initiative sought to explore and document the landscape of One Health resources across rural southeastern Georgia counties, guided by three primary questions: (1) What One Health resources are available in these rural counties? (2) How are these resources accessed by the community? and (3) How can these resources be leveraged to improve workforce development in the area? To address these questions, the project employed PAM to create individual interactive maps highlighting One Health resources in each targeted county. Additional objectives of the initiative included building and strengthening relationships with local community partners and developing county-specific reports. The reports offered key insights and recommendations for workforce development, grounded in One Health. The objective of this paper was to describe the methods used to identify, map, and analyze One Health resources in rural communities through a community-engaged, strengths-based approach.

## 2 Methods used by the Community Health Resource Project

### 2.1 Settings and participants

The state of Georgia has geographic characteristics that include wetlands, mountains, beaches, and swamplands. In the rural regions of the state, several agricultural, manufacturing, and food processing industries drive local economies with natural and historic landmarks bringing new residents and tourists to the area (44, 45). Each county in the state features distinct biodiversity and cultural identity, contributing to a variation in health needs and demographics. For project selection, rural counties in Georgia with populations less than 50,000 were considered for inclusion, as indicated by the State Office of Rural Health (46).

The United States Census Bureau 2019–2023 Community Survey estimates for rural Georgia counties indicated a total population of 2,373,541 people, representing approximately one-fifth of the total population in Georgia. During the same five-year period, 67.14% of individuals identified as White, 23.85% as Black, 0.91% as Asian, 0.33% as American Indian or Alaskan Native, 0.08% as Native Hawaiian or Pacific Islander, 2.43% as belonging to another race, and 5.26% as identifying with two or more races. Approximately 6.75% of the population were of Hispanic or Latino origin. The rate of mental health providers for rural counties in Georgia was 93 per 100,000, in comparison to 191 per 100,000 for the state of Georgia and 333 per 100,000 for the United States. Primary care physicians averaged 76 per 100,000 in the United States, 66 per 100,000 in the state of Georgia, but only 41 per 100,000 in Georgia rural counties. In selected rural counties, health behaviors and factors contributing to adverse health outcomes that were at least two times higher than the national averages included sexually transmitted infections, adult obesity, children in poverty, and injury-related deaths (47, 48).

These indicators were valuable in not only understanding each population uniquely, but also allowed CHRP to better recognize the strategies employed by local organizations to supplement the lack of resources in the region. Thus, workforce development and training strategies influenced by the community conversations were included in reports at the culmination of county outreach efforts to inform future interventions. These strategies were tailored to amplify the strengths of the community, the voices of community members, and the efforts to promote the overall health and well-being with the available resources at their disposal.

### 2.2 Project design

Participatory Asset Mapping was used to guide engagement strategies with community partners during program implementation with target rural counties in southeast Georgia. Asset Mapping, a research method developed by Kretzmann and McKnight, is a strategy for capacity building and community development (49). PAM, a version of Asset Mapping, uses the asset-based approach to gather and analyze the data with the participation of the community (50). The outcomes of PAM are used to encourage interventions influenced by community-driven knowledge and resource mapping (50).

**Figure 1:**
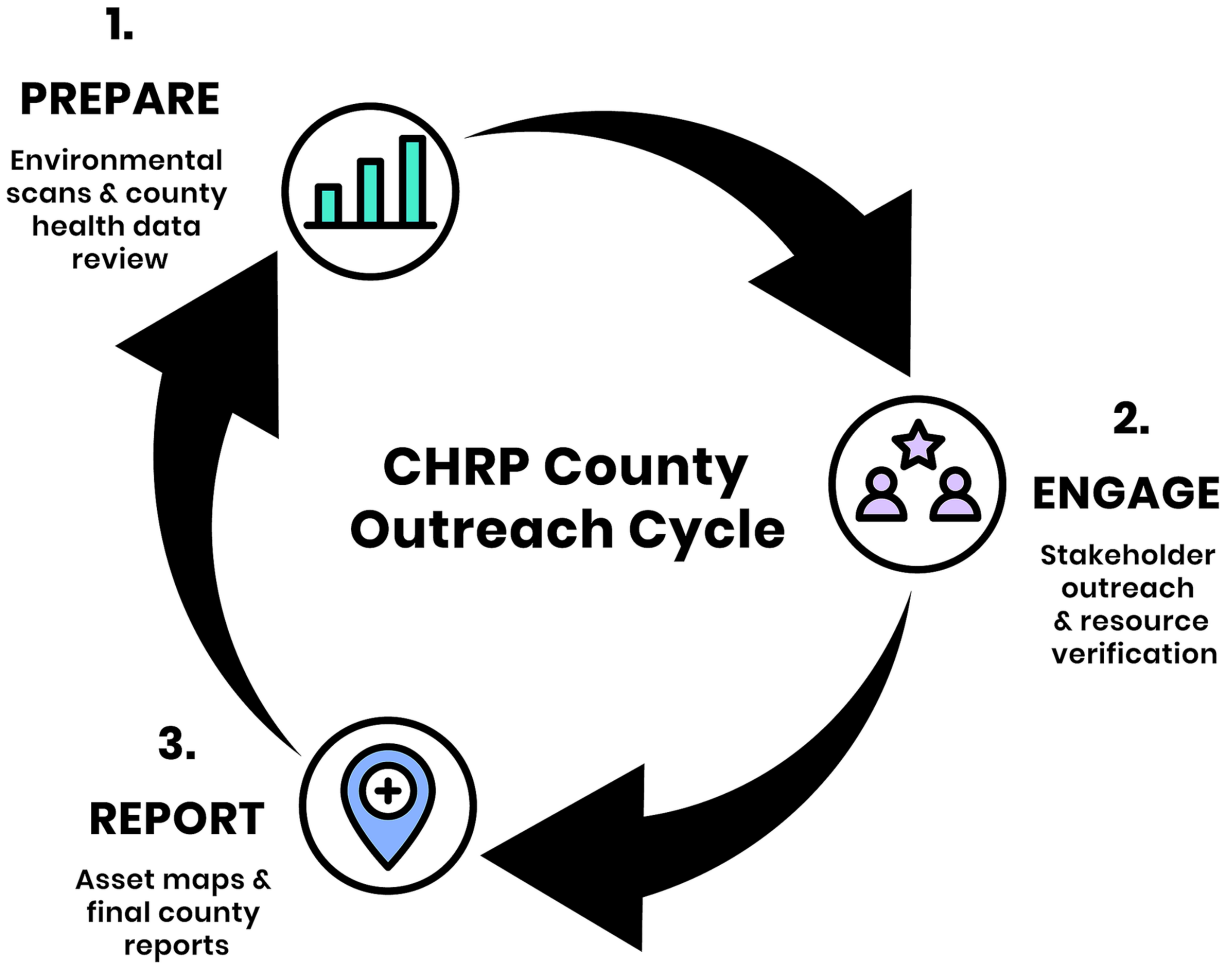
The CHRP “Prepare, Engage, Report” process. The team employed a cyclical “Prepare, Engage, Report” approach in each county over a two-month period. During the *Prepare* phase, team members conducted environmental scans and compiled county health demographic data to contextualize outreach. In the *Engage* phase, the team connected with community stakeholders using various outreach methods to identify and verify One Health resources. In the *Report* phase, findings were documented through interactive maps and final reports, capturing both quantitative and qualitative insights to inform county-specific workforce strategy recommendations.

In an asset-based community development strategy, the community leads the process in exploring their pre-existing assets, mapping them, and referring to those mapped assets to address community issues for community-driven solutions (50). The CHRP team relied on PAM to focus attention on the strengths in the counties. In an effort to adhere to the predetermined timeline of the project, the team followed a “Prepare, Engage, Report” process to gather qualitative and quantitative data through publicly-available datasets and semi-structured interviews with community stakeholders in each county (Figure 1). The “Prepare, Engage, Report” process is iterative by design. Each phase was adapted to each county under the direction of county partners. Team members refined their approach as challenges with engagement and reporting required more preparation for an accurate portrayal of county-specific norms and values. This process allowed for each county-specific report to accurately describe the approach of working with 18 rural counties in southeast Georgia during a two-year period.

The team utilized a strengths-based approach to guide the design “Prepare, Engage, Report” (PER) process. Using CHNAs as an example of needs/deficits-based initiatives, the team adapted PAM and its capacity building and community development constructs for engagement with county residents (5, 15–17, 19, 20). As part of the CHRP design, the team researched which strategies were commonly implemented in rural areas. Studies and interventions that focus on rural health tend to factor morbidity and mortality rates when developing health solutions for this population as it’s important to understand risk factors when engaging with rural communities as a means to provide targeted solutions (5, 14–16). CHRP gathered health statistics of each county to understand the disparities, but used PAM to develop informative reports which highlighted strengths and opportunities.

Six rural counties were determined as pilot sites based on proximity to the university campus. These pilot counties solidified the PER process for rural community outreach strategies used by the CHRP team. The iterations of the PER process were based on feedback from rural community members during outreach. The finalized process provided more streamlined outreach and engagement strategies in the remaining twelve counties. In all counties, preliminary county-level health data was extracted, which revealed indicators of chronic disease burden, mortality, social determinants of health, and access inequities. These health indicators were included at the beginning of each county specific report. Before physically visiting the county, the team engaged key community stakeholders from the county launch. These individuals were selected based on their deep understanding of local health data, existing public health infrastructure, prior engagement in community initiatives, and a reputation for partnership with organizations in the pilot county of the project. During the initial meetings, the team presented the “Prepare, Engage, Report” process and project objectives to the stakeholders. They advised to adapt each “Prepare, Engage, Report” process to reflect each county’s socio-cultural environment. During outreach with each county, stakeholders from different One Health entities were engaged and valued at each stage of the process. County members from different agencies provided input on the CHRP process and deliverables.

Through conversations with county members, the CHRP team routinely evaluated their engagement and reporting strategies throughout the project. For example, one county resident mentioned accessibility during the pilot phase, noting the online asset maps may deter use from individuals who do not rely on technology as much as others. Thus, a PDF list of verified resources was developed and shared with county partners for offline use. In other conversations, community members shared best practices during each county’s engagement phase. Some counties valued initial conversations with those in elected and city positions, while others preferred a more grassroots approach with community volunteers or long-term residents with family ties to the county. This participatory approach was routinely incorporated throughout asset identification and mapping. For example, some resources opted out of being highlighted on county maps, while others clarified services and self-selected their One Health domain.

#### 2.2.1 Prepare

Prior to engaging with key stakeholders, such as community members, county-specific organizations, and elected officials, the team conducted environmental scans of each county. These scans included gathering information on county-specific organizations via social media, the internet, local news stations, and conversations with the CHRP team who may have personal connections to the county. As a part of the environmental scan, the team also created a county health demographic document that included publicly-available health statistics in each county. These statistics were primarily drawn from County Health Rankings for a comprehensive understanding of key county health indicators relative to the state and the nation (51). An initial list of organizations that operate in one of nine broad One Health categories, such as places of spiritual significance, animal/veterinary services, hospitals/clinics, social service support organizations, and parks and recreational services, were gathered. The team implemented both an upstream and downstream approach, compiling a list of elected officials, county government, and Chambers of Commerce, as well as organizations such as Georgia Family Connection, Georgia Public Libraries, United Way, and University of Georgia Cooperative Extension, to name a few. The preparation phase allowed for team members to form an initial understanding of the cultural, historical, and societal norms unique to each county.

#### 2.2.2 Engage

To verify the existence, location, and utility of targeted assets, the CHRP team employed various community engagement methods (52, 53). These methods included emails, calls, text and social media messages, unscheduled drop-in visits to community organizations, scheduled in-person and virtual meetings, attendance at community events, and in-person windshield/walking tours in different parts of the targeted counties. These activities were designed to elicit insights from county partners regarding a wide variety of local health-related assets that may or may not have been identified in the “Prepare” phase. Each engagement method was guided by semi-structured qualitative interview questions, adapted from the National Association of County & City Health Officials’ Mobilizing for Action through Planning and Partnerships Handbook 2.0 (54). The interviews were grounded in strengths-based strategies, fostering conversations that leveraged community expertise to draw attention to local resources. Existing relationships were used in two ways, to assist with project progress and to engage in county trust-building. In 10 of the 18 counties, team members leveraged personal and professional connections to begin highlighting assets and determine county strengths. In later counties, community members from counties that completed the PER process leveraged their connections with the team to introduce additional organizations. Team members focused on relationship building and made clear to county residents and employees the importance of community member expertise, which increased trust and confidence in the project objectives. Using conversations with county members as a guide to direct the engagement process, the team noted that some counties valued initial interactions with elected officials and key influentials whereas other counties valued a more grassroots approach, as seen in the “Prepare” phase. The benefit of these conversations, or qualitative interviews, were made evident by the connections facilitated by community members to other in-county One Health resources garnered through those conversations. During windshield/walking tours, pictures were taken of the social environment for the county-reports. Once resources were verified by community members, the team compiled that information for reporting. Focused engagement with each county was limited to approximately 2 months to ensure timely project reports. In an effort to maintain relationships and trust, the team communicated the end of outreach with community partners via the aforementioned community engagement strategies. Should community members want to update their reported resources or recommend additional resources after the focused engagement period, they were provided with a link to a community questionnaire. After each in-person conversation with county residents and employees, the CHRP team provided a reusable shopping bag and a postcard with contact information as a thank you for their time and participation.

#### 2.2.3 Report

**Figure 2:**
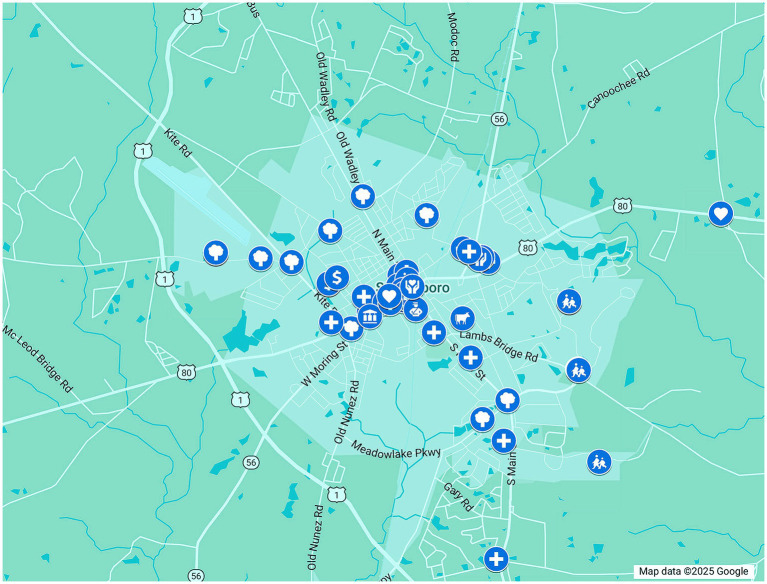
A screenshot of a portion of the Emanuel County asset map created using Google maps. The map displays verified community health resources across Emanuel County, Georgia, using icons to represent various One Health asset domains. Each icon is interactive, allowing users to explore detailed information about individual resources.

Upon completion of community engagement activities, CHRP team members utilized a standardized Google Forms survey, referred to as the *CHRP Reporting Form*, to systematically quantify outreach efforts and capture qualitative insights. This form facilitated the internal documentation of key findings such as effective engagement strategies, community recommendations for enhancing existing resources, and challenges encountered during project implementation in the targeted county. The specific One Health resources gathered and verified during the community engagement phase were entered in another standardized, internal Google Forms survey, referred to as the *CHRP Assets Form*. The highlighted resources were organized by county and One Health category. Following data entry, resource information was extracted to a spreadsheet and spatially visualized using Google My Maps, allowing for the geographic representation of One Health assets by county (Figure 2) (55). This mapping facilitated the identification of service distribution patterns and potential gaps in local health infrastructure. Every county map included a geographic outline of the county with resources organized based on the confirmed address on the interactive map. To showcase the diversity of each resource, each entry was assigned a primary One Health category with the option to choose multiple secondary categories. In addition to the asset map, the CHRP team developed county-specific reports, which compiled all the findings derived from community engagement efforts. The reports included a summary of interviews, observations, and photographic documentation of the understanding of One Health resources in rural southeast Georgia. These reports were intentionally designed to be visually engaging and accessible for a broad range of stakeholders, including community members. Written in clear, straightforward language and organized with user-friendly layouts, the reports prioritized ease of understanding and practical use. Each report featured iconography and visually intuitive charts illustrating key county-level demographic and health statistics, as well as photographs documenting engagement activities with community members. An “Outreach at a Glance” section visually summarized the outreach metrics used in each county. Additional sections analyzed the strengths and challenges of the engagement process, relevant historical and cultural context that informed the identification of One Health assets, and actionable workforce strategies and recommendations. A categorized inventory of verified One Health assets was included, along with a QR code linking to the interactive asset map for ongoing access and updates. These reports were designed to serve as both a practical tool for planning and a recognition of community resilience, emphasizing strengths and opportunities over deficits.

### 2.3 Staff training

As an interdisciplinary team, CHRP recognized each team member brings different perspectives to the project and experiences different challenges when navigating through communities. To establish standard operating procedures (SOPs) while affording space for unique strengths and professional development, the team took a three-pronged approach to staff training. This included project lead training, individual team member training, and comprehensive team training. First, the project manager held weekly one-on-one meetings with the project lead where project management skills, potential team issues, and project methodologies were discussed. Second, the project lead held weekly one-on-ones with each individual CHRP team member where challenges and roadblocks were discussed. Team members were encouraged to share their challenges with their teammates to leverage the diverse strengths of the team. If internal solutions and support were not readily available, team members could access various skill-based training modules housed on the university’s training portal. Finally, the team held weekly staff meetings facilitated by the project lead to discuss county-specific progress. In these staff meetings, training exercises were often conducted to check for biases, collectively solve problems as a team, and enhance the overall skill set of the team members, ensuring consistency across reporting was maintained. These discussions often guided what ultimately was included in the county and final reports.

The team experienced positive interactions with county members who were excited about the project, as well as interactions that required discussions within the team regarding continued engagement and strategizing strengths-based reporting. CHRP reports use grounded theory, and the team utilized team discussions to highlight county residents’ perspectives for reports. Many of these discussions centered on workforce development, recognizing this as a key opportunity for impact. Each strategy combined the strengths-based approach by highlighting what was going well in each county while also providing recommendations on One Health resource growth and sustainability through evidence-based practices. As the project progressed, SOPs were updated to reflect protocol changes further embedding these training protocols into the fabric of the project.

### 2.4 Data collection

**Figure 3:**
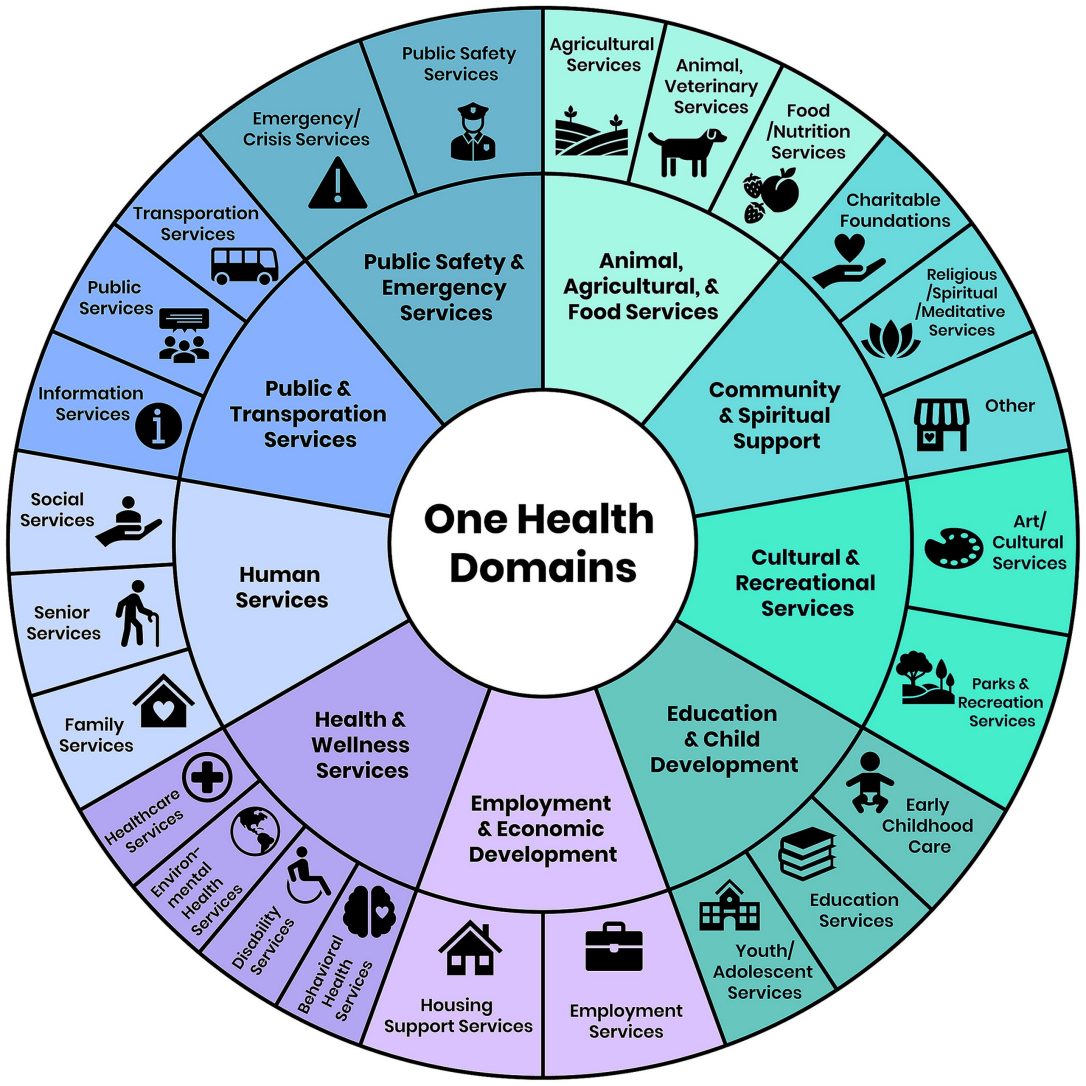
Hierarchical visualization of community resources using One Health domains. This figure presents a two-level categorization of resources identified through PAM. The inner ring depicts the nine primary One Health categories, while the outer ring shows the corresponding secondary classifications. Each subcategory is represented by an icon to facilitate interpretation and highlight the interconnectedness of resources across sectors relevant to One Health.

Quantitative and qualitative data throughout the program was collected using the *CHRP Reporting Form* and the *CHRP Assets Form* completed by the CHRP team members after the county engagement period concluded. The *CHRP Asset Form* was employed to categorize and organize identified resources according to One Health domains (Figure 3). Information was gathered about each asset’s name, verified asset address, One Health asset domain, contact information, and any unique services offered at this location using the One Health Asset Mapping Interview Guide (Supplementary File 1). The *CHRP Reporting Form* was used to document any CHRP activities in the county by CHRP team members, including targeted county, data of activity, type of activity (e.g., outreach, key insights, or other), and type of engagement used (e.g., phone calls, email, community event, meetings, focus groups, windshield/walking tour, drop-ins, and media interactions). Open-ended questions were also available for use to describe the activity in more detail, highlight moments that went well, document moments that did not go well, as well as an opportunity to describe recommendations stated by or interactions encountered with community members that will help address health–related workforce development efforts in their county.

Additional data were also collected using the community-facing survey when community members had additional assets they wanted to report. Due to the iterative nature of CHRP, grounded theory was used to guide the development of workforce strategies and recommendations included in final reports. In qualitative research, grounded theory utilizes inductive reasoning during data collection to allow for common themes during the data analysis and comparison process to emerge, which directly informs the team of the unique experiences of the population of focus (56). The program evaluation of CHRP was reviewed by the Institutional Review Board and determined to be exempt from further review (#H25257).

## 3 Discussion

The Community Health Resource Project’s use of strengths-based outreach strategies allowed each county-specific asset map and report to be informed by and developed through community-led conversations. Participatory Asset Mapping in each county facilitated the spotlighting of One Health resources that community members were able to verify and recommend to be included in the asset maps and reports. After reports were generated and distributed back to each county, community partners were encouraged to provide feedback. This continued the collaborative nature of the project where community members recognized their ability to influence the development of health priorities in their area. By amplifying the voices of community members, the project not only gathered valuable qualitative insights but also cultivated trust and collaboration among community members. The participatory approach underscored that the work was conducted alongside communities, rather than on them, acknowledging that CHRP team members are not the “experts” of the community. This perspective aligns with current literature that reiterates the importance of community involvement in health initiatives, especially in underserved areas (28). It’s common to perceive a lack of resources and opportunities as a deficit, particularly in rural areas. However, by focusing on the community’s strengths and existing assets, it can reveal the hidden potential of these valuable resources, empowering community members to leverage the assets to solve community problems, as well as allow communities to grow themselves (57). This finding was observed during CHRP’s reporting process, where county members responded to the final reports by stating they would use the findings to develop and implement strategies for a path forward in their own community.

### 3.1 Opportunities encountered

Through data collection and in-depth engagement with the counties, there were certain community One Health themes that emerged. These themes were translated into workforce development strategies. For example, the need for cohesive emergency preparedness and response efforts, substance misuse and harm reduction programming, and large and small animal veterinary services were the three most common strategies recommended to county partners. These recurrent topics offer opportunities for further collaboration, leveraging existing community strengths to allow for specifically-tailored, community-informed initiatives. At the end of outreach in all 18 counties, a county-wide community meeting was scheduled to elicit more feedback through the member checking process to discuss these strategies and results from the project.

#### 3.1.1 Rural emergency preparedness and response

It is estimated that because of rising global temperatures, weather events will increase in frequency and severity (58). The first year of CHRP efforts coincided with several major weather events in the Southeast, including Hurricane Helene, which caused significant damage in Georgia (59). During CHRP outreach, many communities were still in the process of recovering from power outages, damaged buildings, and other destruction from fallen trees. Unsurprisingly, emergency preparedness and response emerged as a prominent concern for many communities. The resilience of rural communities after this hurricane was evident through conversations with many shared stories about individuals clearing the roads for their neighbors or providing food for residents without power. In addition to the efforts from public safety and emergency management organizations, it was clear that faith-based and other community-based organizations worked together to provide relief and recovery efforts for their community. Collaborative efforts, as seen during outreach efforts in CHRP counties, are essential for disaster resilience in rural communities (60). By leveraging the strengths and resiliency of these community members and organizations, there are opportunities to grow sustainable and cohesive emergency preparedness and response programs through non-traditional avenues specifically for rural communities, including using social media for response efforts and community-based disaster exercises (61–63). To prevent or mitigate effects of weather events specifically, it is crucial to have effective emergency preparedness and response strategies in place (64). Examples of some of the workforce development strategies recommended to CHRP counties were to (1) build upon existing partnerships and collaborative efforts to conduct both social and geographical risk assessments, (2) implement preparedness training, (3) create a cohesive and comprehensive emergency and disaster response plan for the county, and (4) build community coalitions that can be called upon for recovery efforts. These recommendations are in alignment with evidence-based practices noted in the literature (60, 65, 66).

#### 3.1.2 Substance misuse and harm reduction programming

Another concern among community members in CHRP counties that emerged was substance use, misuse, and addiction. These findings aligned with existing literature, which recognizes that substance misuse, particularly opioid use, has contributed to substantial morbidity and mortality in both metropolitan and rural areas across Georgia (67). Additionally, it was noted that among individuals with opioid use disorder (OUD) in rural areas, there was an increase in methamphetamine use as well (68). In recent years, other substances, such as fentanyl, were found to be increasingly present in both opioids and stimulants, both knowingly and unknowingly (69, 70). While rural areas do not have the resources for individuals with substance use disorder (SUD) that metropolitan areas do (71), the CHRP team was able to draw attention to several resources throughout the counties. Resources such as organizations providing residential and/or medication-assisted treatment (MAT) programs, support groups for individuals in recovery, and alcohol and other drug prevention initiatives and/or coalitions were highlighted. There were also conversations with public safety and first responders who indicated they routinely carry naloxone to reverse an opioid overdose when needed. By building on these existing resources and partnerships, additional harm reduction strategies, such as increased access to naloxone and fentanyl test strips, can be implemented to prevent further morbidity and mortality from OUD (72). Further, programs to increase knowledge and address stigma related to SUD should be included to maximize the effectiveness of harm reduction efforts (73). CHRP’s recommendations to counties consisted of expanding existing community-driven services for individuals with SUD. Leveraging telehealth services to connect individuals to appropriate treatment and support and the implementation of Recovery Community Organizations (RCO) is an example. While the literature supports the use of telemedicine for MAT, access to this technology consistently was one of the barriers noted in rural areas (74, 75).

#### 3.1.3 Large and small animal veterinary services

Counties in the CHRP programmatic region had several agricultural entities, such as farms, dairies, orchards, and hatcheries. Many of these entities provided goods to local farmers’ markets and shops, as well as contributed to Georgia’s overall agricultural export portfolio. Despite several agribusinesses serving Georgia and beyond, interactions with veterinarians revealed a gap in large and small animal-related veterinary services. Conversations in rural counties highlighted a need for large animal veterinarians to support farming operations, with one clinic staff member noting people traveling upwards of two hours to access services. In another county, one community member specifically mentioned a local market as a resource for small animal vaccinations once a month, the only service for companion animals in the area. To address this gap and support the local community, it is essential to develop targeted workforce pipelines to enhance the availability of large and small animal health professionals in rural communities. While transportation barriers in rural areas lead to delayed care and treatment for humans, individuals with companion animals experience the same issues (76). In a study conducted by Smith et al., participants’ interest in telemedicine for veterinary care increased due to the COVID-19 pandemic (77). This finding draws attention to opportunities for rural counties with limited veterinary resources to explore telemedicine options for routine preventative care for animals.

### 3.2 Challenges encountered

Engagement with the counties presented several challenges during the project, primarily due to local dynamics and unforeseen circumstances. While drop-in visits and in-person meetings were the most effective outreach methods and provided valuable insights when visiting rural counties, they were very time-consuming. With only 2 months devoted to county engagement, the limited timeline meant the ability to explore, map, and verify assets were limited, potentially leading to incomplete reporting and possible gaps in our understanding of the communities. Limited time was identified as a challenge given that trust-building in rural communities takes a great while due to high socioeconomic deprivation, stigma, and mistrust of academic institutions (28). Longer and more flexible engagement periods in each county will be essential for fostering deeper relationships and a more comprehensive understanding of community assets.

Another significant challenge encountered during the project was difficulty in reaching and characterizing specific community resources, particularly those associated with faith-based organizations. Hard-to-reach populations are usually floating populations and socially invisible (78); however, this is not usually the case with churches in each county. Several county stakeholders stated that churches and other faith-based groups serve as vital resources within their communities, often offering support and services that contribute to overall community health. However, logistical barriers such as restricted office hours and the limited availability of staff during weekdays impacted outreach efforts with these organizations.

The time of the year the county engagement phase started also impacted the reach in each county. For example, the holiday season or summer months led to slower response rates from county partners. In counties where an organization would normally be included in initial outreach, others required more time building a physical presence in the county for trust building and snowball sampling to take effect. The team would use these moments to revise outreach strategies for successful engagement during the time allotted. As a result, the breadth of resources may have not been fully captured, further contributing to gaps in understanding the overall health landscape in the counties.

Moreover, inclement weather events in the region (hurricanes and winter storms) disrupted outreach efforts in several counties, hindering some in-person interactions with community members in a few targeted counties. These challenges emphasized the need for flexibility in project timelines and methods, as well as the importance of having contingency plans in place to adapt to unforeseen circumstances (79).

### 3.3 Limitations

Several limitations were present in this project. First, the CHRP community outreach team has similar educational backgrounds in public health. While this is helpful for community outreach and engagement, this homogeneity prevents a truly interdisciplinary approach when identifying One Health resources in each county. Although reflexivity was employed to enhance transparency and critical self-awareness throughout the research process, it also presents limitations. The interpretations of findings were inevitably shaped by the research team’s positionalities, professional backgrounds, and prior assumptions (80). The number of social and human service-centered resources far exceeded the resources focused on agricultural, environmental, and animal services in each county. As a way to mitigate selection bias, the team re-oriented project objectives through reflexivity and attempted to prioritize resources in identified One Health categories before new counties were launched.

The CHRP team made valuable connections with several organizations in each county. Organizations such Georgia Family Connection, Georgia Public Libraries, University of Georgia Cooperative Extension, Chambers of Commerce, and City Governments were always included in the initial engagement phase for each county. Although this snowball sampling was successful with building trust and making connections in each county, it led to a tendency for generalizability in the resources highlighted in the project. This limitation with snowball sampling, as Woodley & Lockard discovered, meant there was “no guarantee that the sample will be representative” and the first contacts served as the “gatekeepers,” who have the potential to select respondents based on their own personal biases (81). The team made efforts to reach several different organizations and resources outside of those organizations to increase the diversity of the community members who participated in the project.

The “Prepare, Engage, Report” process presented the CHRP team with limitations as well. Maintaining the strengths-based approach during each phase of the “Prepare, Engage, Report” process excluded the addition of some publicly available resources. Some community members had knowledge of national/statewide hotlines or resources, but utilized local resources in their county instead. However, the use of online resource hubs for rural counties may provide support when local resources are unavailable. During the “Prepare” phase, team members conducted environmental scans of each county. Resources identified during county preparation were then verified and highlighted by county stakeholders. Using the strengths-based approach as a guide translated to the reporting of One Health resources that were verified and recommended by county stakeholders. Although this method empowers community members to leverage their knowledge for community health solutions, the project is cross-sectional by nature (25, 82). Conversations with county residents revealed that frequent staff turnover and inconsistent funding make it challenging for organizations to maintain services. These conversations were similar to a strengths-based study in England which noted that participants who utilized this approach saw challenges with long-term implementation due to community-based organization uncertainty (82).

Finally, the duration of time designated for each county prevented more outreach and relationship building from taking place for the purposes of reporting, Thus, several One Health resources that were harder to reach were excluded from reporting. The lack of time in the counties limited truly systematic qualitative research, as studies have shown researchers build credibility by engaging over extended periods and persistently observing to gain comprehensive insights (83). In rural counties, barriers such as access, transportation, geographic dispersion, and stigma impede residents from being leaders in rural-specific research and workforce development strategies (27). These barriers also presented a challenge for the CHRP team. Engaging with these communities as outsiders required a multi-faceted approach to building trust and relationships in and across counties. Despite these efforts, the team experienced these barriers in some counties more than others, which promoted a more intentional engagement process with One Health agencies. Some organizations had a stronger presence in some counties versus others, and the timeline simply served as a guide for the team to deploy different outreach strategies. However, recognizing this limitation, the CHRP team developed a community-facing survey to reach organizations after outreach concluded in each county.

### 3.4 Implications

CHRP laid a strong foundation for future research and practice by identifying critical needs in community outreach efforts. While the concept of One Health is not new, it has gained significant importance recently, particularly in rural communities that have a strong agricultural presence (84). The outcomes from CHRP showcased the need for targeted strategies that facilitate collaboration with agricultural and environmental organizations in rural areas, which are often overlooked or challenging to engage. Supporting this finding, a study by King et al. emphasized effective outreach strategies, including the principle of “meeting farmers of where they are” (85). This approach advocates for the use of recruitment methods such as placing leaflets in locations where farmers are likely to gather, such as gas stations, sports clubs, and religious organizations, reinforcing the importance of accessible and context-sensitive outreach efforts (85). Ultimately, by prioritizing specific outreach strategies that resonate with rural communities and leveraging existing networks, future research and practice can enhance the effectiveness of One Health initiatives, resulting in a more resilient agricultural industry and better health outcomes across sectors.

Building on this foundational understanding, One Health, more specifically One Rural Health, research can be an integrated approach to rural health research in the 21st century (42). This concept not only addresses the multifaceted health challenges faced by rural populations, but it also draws attention to the pressing need to further explore One Health initiatives within rural health contexts (42). By expanding the scope of research to include diverse populations and settings, health professionals can better understand how to leverage existing resources and foster collaboration across sectors. Integrating the One Health framework can provide valuable implications for future community practice, research, and policy development. This strategy encourages holistic solutions to health challenges by promoting transdisciplinary collaboration between diverse experts, such as physicians, veterinarians, environmental scientists, community organizations, and policymakers (86), especially in areas with limited resources. Collaborative One Health initiatives can lead to shared resources, enhanced workforce development, and the establishment of integrated health programs that support the health and well-being of the community as a whole.

The strengths-based approach used in this project also has significant implications for community practice. By centering the perspectives of community members and highlighting the assets that already exist within the community, this approach fosters empowerment and ownership, allowing space for community members to offer insights and lead the project in unanticipated ways (87). Community members were actively involved in the process of identifying community assets and helped shape the workforce development strategies for each county-specific report. The asset map promoted community collaboration, often by assisting community members and organizations in finding resources they can use to strengthen their own health, work, and/or networks. This participatory approach, which emphasized community strengths and fosters trust, was supported by a study by Kirk et al. that highlighted how top-down, deficit-based, and isolated methods failed to generate the broad-based efforts needed to truly transform communities (19). This approach also has implications for improving trust and collaboration between community members and external partners. As the project demonstrated, when community members were involved in the decision making and asset identification, they were more likely to trust the process and actively engage in the initiative as seen in other research (88). This approach enhanced future community-health interventions by ensuring initiatives were community-driven and rooted in the community’s own values and needs.

Rural, underserved counties in southeast Georgia are experiencing drastic changes. Several ports, shipping warehouses, and vehicle production facilities are being built in unincorporated areas within the counties. Population growth and changing demographics challenge the rural infrastructure that once met the most immediate needs of the community. However, the frequency of severe weather phenomena and its impact on agricultural, environmental, and social well-being are taking a toll on these communities (80, 89, 90). According to Dewi et al., extreme weather events impact health by both directly and indirectly raising illness rates and altering patterns of healthcare utilization (80). These events tend to be pronounced in rural and remote regions, which intensifies existing health disparities and requires immediate strategies for mitigation or adaptation. Although the CHRP approach promoted the scaffolding of pre-existing resources in rural counties and empowered community members to provide successful solutions, incorporating One Health principles has the potential to assist limited resource settings to maximize public impact.

## 4 Summary

The Community Health Resource Project implemented a “Prepare, Engage, Report” process in 18 rural counties in southeast Georgia over a two-year period. Before interacting with each county, the team prepared their engagement strategy by conducting environmental scans to have a better understanding of the health indicators and social dynamics. The team also searched for key stakeholders that would not only facilitate snowball sampling, but would also initiate the trust building process within each county. The team utilized a strengths-based approach throughout the project, highlighting what community members and organizations are doing well within their counties, to identify a broad base of One Health resources. While applying outreach strategies, such as unscheduled drop-ins and scheduled in-county meetings, workforce development themes emerged and the team was able to provide targeted recommendations through county-specific reports that built on existing resources. Effective community engagement for One Health asset mapping in rural communities required participatory approaches that positioned county partners as content experts at each phase of project implementation. This approach ensured culturally appropriate methods were used and actionable strategies that spanned human, animal, plant, and environmental health were identified.

## Data Availability

Publicly available datasets were analyzed in this study. This data can be found at: https://www.census.gov/en.html; https://www.countyhealthrankings.org.

## Supplementary material

SUPPLEMENTARY FILE 1 Interview and focus group guide adapted by the CHRP team during engagement and outreach. The guides were to facilitate strengths-based conversations to understand county-specific, One Health resources in rural communities.
