# Supplementary material for: Community health resource project: highlighting One Health resources across rural Georgia to build healthier communities
Source: Front Public Health. 2025 Nov 6;13:1619886. doi: 10.3389/fpubh.2025.1619886 (PMC12631809; doi:10.3389/fpubh.2025.1619886)
Supplement: SUPPLEMENTARY FILE 1 — Interview and focus group guide adapted by the CHRP team during engagement and outreach. The guides were to facilitate strengths-based conversations to understand county-specific, One Health resources in rural communities. [file Data_Sheet_1.pdf]

One Health Asset Mapping Interview Question Guide

Developed by The Institute for Health Logistics & Analytics.

How to Use This Guide

The use of this interview and focus group guide is intended to support strengths-based community outreach efforts to understand One Health resources in each of the counties. This guide is best used during the "Engage Phase" of the project. Before each community interaction, take time to understand the county and its residents during the Prepare Phase. Information gathered during the "Prepare Phase" can be used to adapt the questions below to promote successful interactions with community members while being mindful of their time and capacity during conversations. After each interaction with the community, log outreach efforts in the appropriate tracking sheet. During the "Report Phase", outreach and engagement will be logged in the project reporting form or the confirmed One Health asset form and used for county specific asset maps and reports.

Interview Questions (by Theme)	
**Contact Information**	• Name, Occupation, Address, Telephone Number, Name of Organization/ Affiliation, Type of Organization/Affiliation
**Community Landscape**	• Please describe the landscape of (X) county.<br>• Is the term "rural" or "underserved" appropriate for (X) county?
**Health Issues**	• Please describe any health issues that negatively impact your county.<br>• Are there any initiatives that help address these issues?
**Organization Role**	• Tell me more about your work/ organization.<br>• What kinds of services does your organization provide to the community? How do you make these services known to the public? What kinds of projects are your organization involved in now? What has your organization accomplished thus far?
**Resources**	• What are your organization's most valuable resources and strongest assets?
**Associations**	• Does your group belong to any other associations?<br>• What kinds of special events does your organization take part in?
**Community Vision**	• What kind of changes would you like to see in the community in the next five years?<br>• How would you effect these changes?

Questions in this Guide Adapted From:

Berkowitz, B., & Wadud, E. (n.d.). *Chapter 3, Section 8: Identifying community assets and resources – Tools. In Community Tool Box.* Center for Community Health and Development, University of Kansas. Retrieved October 3, 2025, from https://ctb.ku.edu/en/table-of-contents/assessment/assessing-community-needs-and-resources/identify-community-assets/tools

National Association of County and City Health Officials. (2022, December). *Community context assessment qualitative assessment tool for Mobilizing for Action through Planning and Partnerships (MAPP) 2.0. In MAPP 2.0 Handbook.* https://www.naccho.org/uploads/resource-hub-images/MAPP-Handbook-Digital-FINAL-Fillable-2025-comp.pdf

Please describe any of the following One Health Assets that exist in this county.	
**Primary Category**	**Secondary Category**
**Animal, Agricultural, and Food Services**	Agricultural, Animal/Veterinary, Food/Nutrition Services
**Community and Spiritual Support**	Charitable, Religious/Spiritual/Meditative Services
**Cultural and Recreational Services**	Art/Cultural, Parks & Recreation Services
**Education and Child Development**	Early Childhood Care, Education, Youth/Adolescent Services
**Employment and Economic Support**	Employment. Housing Support Services
**Health and Wellness Services**	Behavioral Health, Disability, Environmental Health, Healthcare Services
**Human Services**	Family, Social, Senior Services
**Public Safety and Emergency Services**	Emergency/Crisis, Public Safety Services
**Public and Transportation Services**	Public (Government), Transportation, Information Services
**Other**	Other

Community Context Assessment Guiding Questions (Focus Groups)	
**Community Strengths and Assets**	• What One Health related strengths do community members have?<br>• What do you consider to be your community?<br>• What do you think are important characteristics of a healthy community?<br>• Which strengths and assets can be used and strengthened to address One Health inequities?
**Built Environment**	• What physical assets and resources exist in the built environment of the community?<br>• How do these resources differ across neighborhoods, particularly in those experiencing the greatest health inequities?<br>• How do community members view and interact with their built environment?<br>• How do these interactions impact community members' health?<br>• What aspects of the built environment in communities impact health inequities?<br>• How can those aspects be addressed to improve community One Health?
**Forces of Change**	• What do you think are the most significant One Health needs in your community?<br>• Do the organizations we highlighted work to solve these One Health problems? How?<br>• What strategies do you think would make these organizations more successful in addressing these problems?<br>• How does historical context shape the forces of change today, and who benefits from current conditions?<br>• How have climate change and COVID-19 changed the community?<br>• What are the strengths in your community that support these strategies? What can prevent these strategies from being successful?
